# Crystal structure of di-μ-chlorido-bis­(chlorido­{*N* ^1^,*N* ^1^-diethyl-*N* ^4^-[(pyridin-2-yl-κ*N*)methyl­idene]benzene-1,4-di­amine-κ*N* ^4^}mercury(II))

**DOI:** 10.1107/S2056989017005874

**Published:** 2017-05-05

**Authors:** Md. Serajul Haque Faizi, Necmi Dege, Kateryna Goleva

**Affiliations:** a Department of Chemistry, College of Science, Sultan Qaboos University, PO Box 36 Al-Khod 123, Muscat, Sultanate of Oman; b Ondokuz Mayıs University, Arts and Sciences Faculty, Department of Physics, 55139 Samsun, Turkey; c Department of Chemistry, Taras Shevchenko National University of Kyiv, 64, Vladimirska Str., Kiev 01601, Ukraine

**Keywords:** crystal structure, binuclear mercury(II) complex, five-coordinated mercury(II) ions, distorted square-pyramidal coordination, DPMBD, Schiff base

## Abstract

The title dinuclear mercury(II) complex, [Hg_2_Cl_4_(C_16_H_19_N_3_)_2_], synthesized from the pyridine-derived Schiff base (*E*)-*N* ^1^,*N* ^1^-diethyl-*N* ^4^-[(pyridin-2-yl)methyl­idene]benzene-1,4-di­amine (DPMBD), has inversion symmetry. The five-coordinated Hg^II^ atoms have distorted square-pyramidal stereochemistry comprising two N-atom donors from bidentate chelate BPMBD ligands and three Cl-atom donors, two bridging and one monodentate. The dihedral angle between the benzene and the pyridine rings in the BPMBD ligand is 7.55 (4)°. In the crystal, the dinuclear mol­ecules are linked by weak C—H⋯Cl hydrogen bonds, forming zigzag ribbons lying parallel to [001]. Also present in the structure are π–π inter­actions between benzene and pyridine rings [minimum ring-centroid separation = 3.698 (8) Å].

## Abstract 2

The title dinuclear mercury(II) complex, [Hg_2_Cl_4_(C_16_H_19_N_3_)_2_], synthesized from the pyridine-derived Schiff base (*E*)-*N* ^1^,*N* ^1^-diethyl-*N* ^4^-[(pyridin-2-yl)methyl­idene]benzene-1,4-di­amine (DPMBD), has inversion symmetry with the five-coordinated Hg^II^ centres having distorted square-pyramidal stereochemistry comprising two N-atom donors from a bidentate chelate BPMBD ligand and three Cl-atom donors, one monodentate and two bridging.

## Chemical context   

Mercury is one of the most prevalent toxic metals in the environment and gains access to the body orally or dermally, causing cell dysfunction that consequently leads to health problems (Mandal *et al.*, 2012 ▸). Schiff base complexes of 2-pyridine­carboxaldehyde and its derivatives have been found to be good herbicides, used for the protection of plants (Hughes & Prince, 1978 ▸). Transition metal complexes of pyridyl Schiff bases have found applications in catalysis (Kasselouri *et al.*, 1993 ▸). Pyridyl derivatives of Schiff bases are important building blocks for many important compounds, widely used in biological applications such as anti­oxidative, anti­cancer agents, as fluorescent probes in industry, in coordination chemistry and in catalysis (Jursic *et al.*, 2002 ▸; Song *et al.*, 2011 ▸; Motswainyana *et al.*, 2013 ▸; Das *et al.*, 2013 ▸). Our research inter­est focuses on a study of Schiff bases derived from *N* ^1^,*N* ^1^-diethyl-*p*-phenyl­enedi­amine and their metal complexes (Faizi & Hussain, 2014 ▸; Faizi *et al.*, 2015 ▸). We report herein the synthesis and the crystal structure of a new complex of mercury(II), [Hg_2_Cl_4_(C_16_H_19_N_3_)_2_], with the pyridine-derived Schiff base (*E*)-*N* ^1^,*N* ^1^-diethyl-*N* ^4^-[(pyridin-2-yl)methyl­idene]benzene-1,4-di­amine (DPMBD).

## Structural commentary   

**Figure 1:**
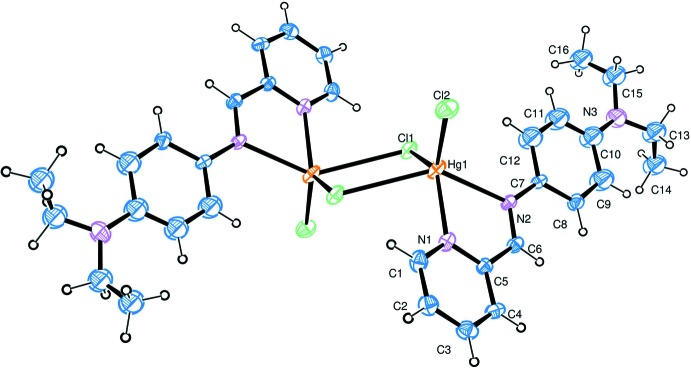
The mol­ecular structure of the title compound, with the atom labelling. Displacement ellipsoids are drawn at the 40% probability level. The unlabelled atoms are related to the labelled atoms by inversion symmetry (symmetry operation: −*x* + 2, −*y* + 1, −*z* + 1).

The dinuclear mol­ecule of the title complex is generated by inversion symmetry (Fig. 1 ▸). The Schiff base-derived ligand (DPMBD) coordinates to the Hg^II^ atom in a bidentate chelating mode through the N atoms of the pyridine ring (N1) and the imine group (N2) [Hg1—N = 2.317 (9) and 2.437 (8) Å, respectively].
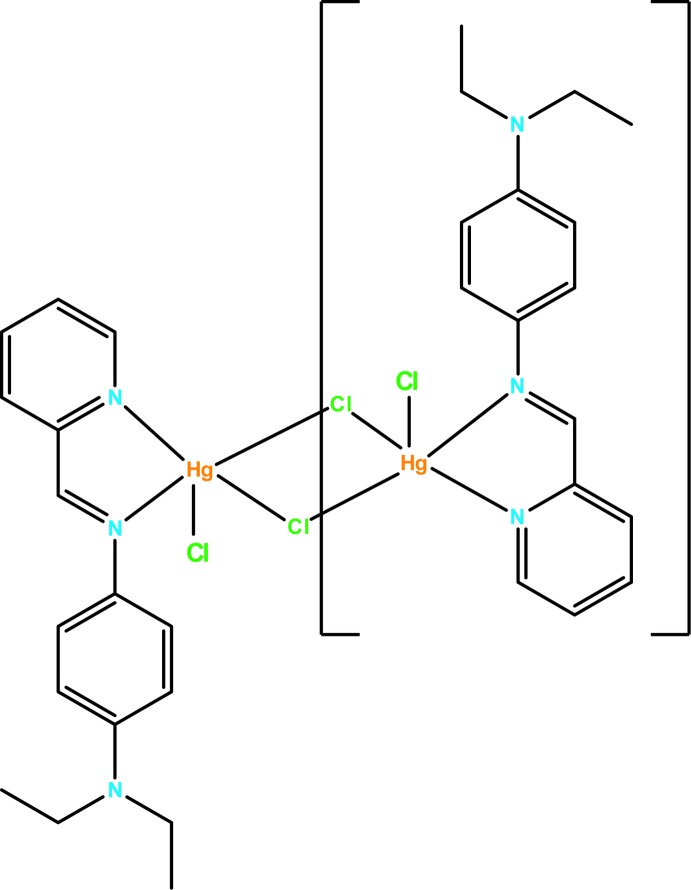


The five-coordinated Hg^2+^ ion has a distorted square-pyramidal geometry completed by three Hg—Cl bonds, one monodentate [Hg1—Cl2 = 2.402 (4) Å] and two bridging Hg1—Cl1 [2.459 (3) Å] and Hg1—Cl1^i^ [2.999 (3) Å; symmetry code: (i) −*x* + 2, −*y* + 1, −*z* + 1]. The environment of a five-coordinated mercuric ion is common among Hg^2+^ complexes (Baul *et al.*, 2004 ▸). The longest Hg—Cl distance bridges across the centre of inversion, giving an Hg⋯Hg^i^ separation of 4.1985 (16) Å. The observed Hg—Cl and Hg—N bond lengths and bond angles are considered normal for this type of Hg^II^ complex (Faizi & Prisyazhnaya, 2015 ▸; Faizi & Sen, 2014 ▸). The benzene and pyridine rings of the DPMBD ligand form a dihedral angle of 7.55 (4)°.

## Supra­molecular features   

**Figure 2:**
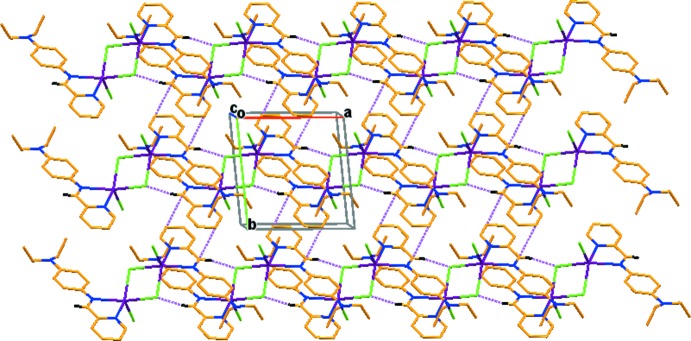
The crystal packing of the title compound, viewed along the *c* axis, with hydrogen bonds (Table 1 ▸) shown as dashed lines.

**Figure 3:**
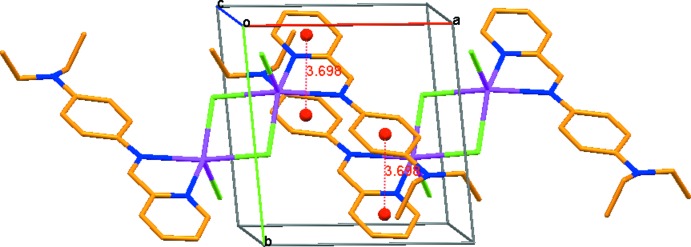
The crystal packing of the title compound, viewed approximately along the *c* axis. The π–π inter­actions between the benzene and pyridine rings are shown as dotted lines.

In the crystal, mol­ecules are linked by C—H⋯Cl hydrogen bonds, forming a sheet like arrangement parallel to [001] (see Table 1 ▸ and Fig. 2 ▸ for details). The centroid-to-centroid distance between inversion-related benzene rings (−*x* + 1, −*y* + 2, −*z* + 1) is 3.879 (6) Å, indicating a weak π–π inter­action along the *c* axis (Fig. 2 ▸). Also present is a benzene–pyridine ring inter­action with *Cg*⋯*Cg* (−*x* + 1, −*y* + 1, −*z* + 1) = 3.698 (8) Å (Fig. 3 ▸).

## Database survey   

A search of the Cambridge Structure Database (Version 5.37 with updates May 2016; Groom *et al.*, 2016 ▸) reveals that there is no entry in the literature for a dichloridomercury(II) complex with (*E*)-*N* ^1^,*N* ^1^-diethyl-*N*4-(pyridin-2-yl­methyl­ene)benzene-1,4-di­amine that has been structurally characterized. A dihalomercury(II) complex has been reported by Baul *et al.* (2013 ▸) in which the Hg^II^ atom is coordinated by the bis-chelating *N*-heterocyclic ligand [(*E*)-*N*-(pyridin-2-yl­methyl­idene)aryl­amine)], two bridging Cl ligands and one terminal Cl ligand. Similar Hg^II^ complexes have also been reported with a slight modification of the ligand (Nejad *et al.*, 2010 ▸), *viz.* di-μ-chlorido-bis­{chlorido­[2-(phenyl­imino­meth­yl)-pyridine-κ^2^ *N*,*N*′]mercury(II)} (Salehzadeh *et al.*, 2011 ▸) di-μ-chlorido-bis­{chlorido­[4-nitro-*N*-(pyridin-2-yl­methyl­idene-κ*N*)aniline-κ*N*]mercury(II)} (Hoseyni *et al.*, 2012 ▸), di-μ-chlorido-bis{chlorido­[2,3-dimethyl-*N*-(pyridin-2-yl­methyl­idene)aniline-κ^2^ *N*,*N*′]mercury(II)} (Faizi & Prisyazhnaya, 2015 ▸) and di-μ-chlorido-bis-(chlorido­{*N* ^1^-phenyl-*N* ^4^)-[(pyridin-2-yl-κ*N*)methyl­idene]benzene-1,4-di­amine-κ*N* ^4^} mercury(II)). All of the above compounds show the Hg^II^ ion in a distorted square-pyramidal coordination environment formed by the N atoms of the di­imine ligand, two bridging Cl atoms and one monodentate Cl atom, as found in the title compound, one of the bridging Hg—Cl bonds being significantly longer than the other.

## Synthesis and crystallization   

The imino­pyridyl compound (*E*)-*N* ^1^,*N* ^1^-diethyl-*N* ^4^-[(pyridin-2-yl)methyl­idene]benzene-1,4-di­amine (DPMBD) was prepared by adding portionwise pyridine-2-carbaldehyde (0.29 g, 2.71 mmol) to a methano­lic solution (50 ml) of *N* ^1^,*N* ^1^-diethyl-*p*-phenyl­enedi­amine (0.50 g, 2.71 mmol). The reaction mixture was stirred for 3 h at room temperature and filtered. The resulting yellow powder was washed with methanol (2 × 3 ml) and hexane (3 × 10 ml). The compound was recrystallized from hot MeOH to give yellow crystals, which were dried in a vacuum desiccator to give the pure product (yield: 0.60 g, 80%).

The title compound was prepared by reacting DPMBD (0.10 g, 0.39 mmol) with mercury(II) chloride (0.05 g, 0.18 mmol) in methanol (5 ml), with vigorous stirring for 2 h at room temperature. The red precipitate that formed was filtered off and redissolved in di­methyl­formamide. Crystals of the red title complex (yield: 0.31 g, 76%) suitable for X-ray analysis were obtained within 3 d by slow evaporation of the di­methyl­formamide.

## Refinement   

Crystal data, data collection and structure refinement details are summarized in Table 2 ▸. H atoms bonded to C atoms were placed in calculated positions with C—H = 0.93–0.97 Å and included in the refinement in a riding-model approximation with *U*_iso_(H) = 1.5*U*_eq_(C) (for methyl H) and *U*_iso_(H) = 1.2*U*_eq_(C) (for other H atoms).

## Supplementary Material

Crystal structure: contains datablock(s) I. DOI: 10.1107/S2056989017005874/zs2377sup1.cif

Structure factors: contains datablock(s) I. DOI: 10.1107/S2056989017005874/zs2377Isup2.hkl

CCDC reference: 1531593

Additional supporting information:  crystallographic information; 3D view; checkCIF report

## Figures and Tables

**Table 1:** Hydrogen-bond geometry (Å, °)

*D*—H⋯*A*	*D*—H	H⋯*A*	*D*⋯*A*	*D*—H⋯*A*
C6—H6⋯Cl1^i^	0.93	2.74	3.578 (9)	151
C1—H1⋯Cl1^ii^	0.93	2.89	3.471 (12)	122
C1—H1⋯Cl2^iii^	0.93	2.97	3.623 (11)	129

Symmetry codes: (i) 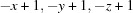; (ii) 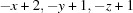; (iii) 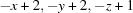.

**Table 2:** Experimental details

Crystal data	
Chemical formula	[Hg_2_Cl_4_(C_16_H_19_N_3_)_2_]
*M*_r_	1049.66
Crystal system, space group	Triclinic, *P* 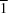
Temperature (K)	100
*a*, *b*, *c* (Å)	8.329 (3), 8.565 (3), 12.936 (4)
α, β, γ (°)	89.043 (8), 81.107 (7), 84.206 (7)
*V* (Å^3^)	907.1 (5)
*Z*	1
Radiation type	Mo *K*α
μ (mm^−1^)	8.78
Crystal size (mm)	0.20 × 0.15 × 0.12

Data collection	
Diffractometer	Bruker APEXII CCD
Absorption correction	Multi-scan (*SADABS*; Bruker, 2003 ▸)
*T*_min_, *T*_max_	0.944, 0.981
No. of measured, independent and observed [*I* > 2σ(*I*)] reflections	6272, 3303, 2779
*R*_int_	0.037
(sin θ/λ)_max_ (Å^−1^)	0.606

Refinement	
*R*[*F* ^2^ > 2σ(*F* ^2^)], *wR*(*F* ^2^), *S*	0.054, 0.156, 1.06
No. of reflections	3303
No. of parameters	158
No. of restraints	57
H-atom treatment	H-atom parameters constrained
Δρ_max_, Δρ_min_ (e Å^−3^)	2.56, −2.43

Computer programs: *APEX2* and *SAINT* (Bruker, 2003 ▸), *SHELXS97* (Sheldrick, 2008 ▸), *SHELXL2016* (Sheldrick, 2015 ▸) and *DIAMOND* (Brandenburg & Putz, 2006 ▸).

## supplementary crystallographic information

### Crystal data

**Table:**

[Hg_2_Cl_4_(C_16_H_19_N_3_)_2_]	*Z* = 1
*M**_r_* = 1049.66	*F*(000) = 500
Triclinic, *P*1	*D*_x_ = 1.921 Mg m^−^^3^
*a* = 8.329 (3) Å	Mo *K*α radiation, λ = 0.71073 Å
*b* = 8.565 (3) Å	Cell parameters from 3407 reflections
*c* = 12.936 (4) Å	θ = 2.7–26.6°
α = 89.043 (8)°	µ = 8.78 mm^−^^1^
β = 81.107 (7)°	*T* = 100 K
γ = 84.206 (7)°	Needle, red
*V* = 907.1 (5) Å^3^	0.20 × 0.15 × 0.12 mm

### Data collection

**Table:**

Bruker APEXII CCD diffractometer	3303 independent reflections
Radiation source: sealed tube	2779 reflections with *I* > 2σ(*I*)
Graphite monochromator	*R*_int_ = 0.037
φ and ω scans	θ_max_ = 25.5°, θ_min_ = 2.4°
Absorption correction: multi-scan (SADABS; Bruker, 2003)	*h* = −10→10
*T*_min_ = 0.944, *T*_max_ = 0.981	*k* = −7→10
6272 measured reflections	*l* = −15→15

### Refinement

**Table:**

Refinement on *F*^2^	Hydrogen site location: inferred from neighbouring sites
Least-squares matrix: full	H-atom parameters constrained
*R*[*F*^2^ > 2σ(*F*^2^)] = 0.054	*w* = 1/[σ^2^(*F*_o_^2^) + (0.0936*P*)^2^ + 2.7873*P*] where *P* = (*F*_o_^2^ + 2*F*_c_^2^)/3
*wR*(*F*^2^) = 0.156	(Δ/σ)_max_ < 0.001
*S* = 1.06	Δρ_max_ = 2.56 e Å^−^^3^
3303 reflections	Δρ_min_ = −2.43 e Å^−^^3^
158 parameters	Extinction correction: SHELXL2016 (Sheldrick, 2015), Fc^*^=kFc[1+0.001xFc^2^λ^3^/sin(2θ)]^-1/4^
57 restraints	Extinction coefficient: 0.0154 (18)

### Special details

**Table:**

Geometry. All esds (except the esd in the dihedral angle between two l.s. planes) are estimated using the full covariance matrix. The cell esds are taken into account individually in the estimation of esds in distances, angles and torsion angles; correlations between esds in cell parameters are only used when they are defined by crystal symmetry. An approximate (isotropic) treatment of cell esds is used for estimating esds involving l.s. planes.

### Fractional atomic coordinates and isotropic or equivalent isotropic displacement parameters (Å^2^)

**Table:**

	*x*	*y*	*z*	*U*_iso_*/*U*_eq_	
Hg1	0.81436 (5)	0.66012 (4)	0.57709 (3)	0.0580 (3)	
Cl1	0.8912 (3)	0.3764 (3)	0.5896 (2)	0.0565 (6)	
Cl2	0.8896 (4)	0.8431 (4)	0.6955 (3)	0.0756 (8)	
N2	0.5206 (9)	0.6438 (8)	0.5996 (6)	0.0418 (16)	
N1	0.7065 (10)	0.8114 (9)	0.4494 (7)	0.0486 (18)	
C5	0.5464 (11)	0.8065 (10)	0.4459 (7)	0.0425 (19)	
C7	0.4285 (19)	0.564 (2)	0.6811 (12)	0.0965 (13)	
C6	0.4541 (11)	0.7127 (10)	0.5239 (7)	0.045 (2)	
H6	0.345407	0.701618	0.519674	0.054*	
C4	0.4711 (14)	0.8919 (12)	0.3733 (8)	0.054 (2)	
H4	0.361040	0.884616	0.371099	0.064*	
C8	0.2645 (12)	0.5445 (14)	0.6857 (8)	0.060 (3)	
H8	0.207736	0.588526	0.634069	0.072*	
C2	0.7167 (15)	0.9953 (13)	0.3116 (9)	0.062 (3)	
H2	0.776639	1.064049	0.268772	0.075*	
C10	0.266 (2)	0.389 (2)	0.8458 (13)	0.1012 (7)	
C1	0.7883 (14)	0.9042 (13)	0.3803 (10)	0.062 (3)	
H1	0.899938	0.905612	0.379875	0.075*	
C12	0.505 (2)	0.498 (2)	0.7587 (12)	0.1012 (7)	
H12	0.613677	0.512565	0.760116	0.121*	
N3	0.1794 (12)	0.3036 (16)	0.9288 (8)	0.1012 (9)	
C3	0.5551 (16)	0.9872 (13)	0.3044 (9)	0.063 (3)	
H3	0.505047	1.044522	0.254433	0.075*	
C9	0.186 (2)	0.462 (2)	0.7646 (13)	0.1012 (7)	
H9	0.075706	0.452541	0.766178	0.121*	
C11	0.424 (2)	0.409 (2)	0.8348 (13)	0.1012 (7)	
H11	0.484613	0.359696	0.882884	0.121*	
C15	0.2584 (16)	0.2780 (17)	1.0150 (10)	0.1012 (7)	
H15A	0.182347	0.272105	1.079438	0.121*	
H15B	0.331868	0.357020	1.021477	0.121*	
C16	0.3500 (18)	0.1193 (16)	0.9831 (12)	0.1012 (7)	
H16A	0.413710	0.082530	1.036072	0.152*	
H16B	0.273224	0.045619	0.975182	0.152*	
H16C	0.420937	0.129507	0.917922	0.152*	
C13	0.0131 (13)	0.3113 (19)	0.9350 (12)	0.1012 (7)	
H13A	−0.023986	0.413409	0.909047	0.121*	
H13B	−0.033164	0.307139	1.008496	0.121*	
C14	−0.059 (2)	0.1883 (19)	0.8782 (12)	0.1012 (7)	
H14A	−0.176271	0.208254	0.889439	0.152*	
H14B	−0.019235	0.192811	0.804657	0.152*	
H14C	−0.028458	0.086028	0.904586	0.152*	

### Atomic displacement parameters (Å^2^)

**Table:**

	*U*^11^	*U*^22^	*U*^33^	*U*^12^	*U*^13^	*U*^23^
Hg1	0.0436 (3)	0.0551 (3)	0.0792 (4)	−0.00681 (18)	−0.0217 (2)	0.0087 (2)
Cl1	0.0404 (12)	0.0548 (13)	0.0761 (16)	−0.0047 (10)	−0.0161 (11)	0.0139 (11)
Cl2	0.0711 (19)	0.0739 (18)	0.089 (2)	−0.0146 (15)	−0.0278 (16)	−0.0072 (15)
N2	0.039 (4)	0.037 (4)	0.052 (4)	−0.007 (3)	−0.010 (3)	0.000 (3)
N1	0.042 (4)	0.041 (4)	0.065 (5)	−0.007 (3)	−0.012 (4)	−0.004 (3)
C5	0.040 (5)	0.039 (4)	0.049 (5)	−0.001 (4)	−0.012 (4)	−0.010 (4)
C7	0.080 (2)	0.124 (2)	0.089 (2)	−0.025 (2)	−0.018 (2)	0.032 (2)
C6	0.039 (5)	0.042 (4)	0.057 (5)	−0.005 (4)	−0.018 (4)	−0.007 (4)
C4	0.056 (6)	0.054 (6)	0.053 (5)	0.000 (5)	−0.022 (5)	0.002 (4)
C8	0.038 (5)	0.087 (8)	0.058 (6)	−0.011 (5)	−0.012 (4)	0.020 (5)
C2	0.073 (8)	0.052 (6)	0.060 (6)	−0.010 (5)	−0.003 (5)	0.007 (5)
C10	0.0849 (13)	0.1288 (13)	0.0928 (13)	−0.0231 (13)	−0.0184 (12)	0.0329 (13)
C1	0.046 (6)	0.056 (6)	0.083 (7)	−0.009 (5)	−0.005 (5)	0.009 (5)
C12	0.0849 (13)	0.1288 (13)	0.0928 (13)	−0.0231 (13)	−0.0184 (12)	0.0329 (13)
N3	0.0848 (16)	0.1290 (17)	0.0927 (15)	−0.0230 (16)	−0.0183 (15)	0.0330 (16)
C3	0.077 (8)	0.049 (5)	0.064 (6)	0.003 (5)	−0.018 (6)	0.002 (5)
C9	0.0849 (13)	0.1288 (13)	0.0928 (13)	−0.0231 (13)	−0.0184 (12)	0.0329 (13)
C11	0.0849 (13)	0.1288 (13)	0.0928 (13)	−0.0231 (13)	−0.0184 (12)	0.0329 (13)
C15	0.0849 (13)	0.1288 (13)	0.0928 (13)	−0.0231 (13)	−0.0184 (12)	0.0329 (13)
C16	0.0849 (13)	0.1288 (13)	0.0928 (13)	−0.0231 (13)	−0.0184 (12)	0.0329 (13)
C13	0.0849 (13)	0.1288 (13)	0.0928 (13)	−0.0231 (13)	−0.0184 (12)	0.0329 (13)
C14	0.0849 (13)	0.1288 (13)	0.0928 (13)	−0.0231 (13)	−0.0184 (12)	0.0329 (13)

### Geometric parameters (Å, º)

**Table:**

Hg1—Cl1	2.459 (3)	C11—C12	1.37 (2)
Hg1—Cl2	2.402 (4)	C13—C14	1.52 (2)
Hg1—N1	2.317 (9)	C15—C16	1.52 (2)
Hg1—N2	2.437 (8)	C1—H1	0.9300
Hg1—Cl1^i^	2.999 (3)	C2—H2	0.9300
N1—C1	1.339 (15)	C3—H3	0.9300
N1—C5	1.346 (13)	C4—H4	0.9300
N2—C6	1.302 (12)	C6—H6	0.9300
N2—C7	1.414 (18)	C8—H8	0.9300
N3—C10	1.43 (2)	C9—H9	0.9300
N3—C13	1.370 (15)	C11—H11	0.9300
N3—C15	1.384 (17)	C12—H12	0.9300
C1—C2	1.342 (17)	C13—H13A	0.9700
C2—C3	1.372 (19)	C13—H13B	0.9700
C3—C4	1.360 (16)	C14—H14A	0.9600
C4—C5	1.370 (14)	C14—H14B	0.9600
C5—C6	1.453 (13)	C14—H14C	0.9600
C7—C8	1.385 (19)	C15—H15A	0.9700
C7—C12	1.36 (2)	C15—H15B	0.9700
C8—C9	1.35 (2)	C16—H16A	0.9600
C9—C10	1.43 (2)	C16—H16B	0.9600
C10—C11	1.33 (2)	C16—H16C	0.9600
			
Cl1—Hg1—Cl2	121.69 (10)	N1—C1—H1	118.00
Cl1—Hg1—N1	131.9 (2)	C2—C1—H1	118.00
Cl1—Hg1—N2	96.06 (17)	C1—C2—H2	120.00
Cl1—Hg1—Cl1^i^	79.90 (8)	C3—C2—H2	120.00
Cl2—Hg1—N1	105.7 (2)	C2—C3—H3	121.00
Cl2—Hg1—N2	112.60 (19)	C4—C3—H3	121.00
Cl1^i^—Hg1—Cl2	102.68 (10)	C3—C4—H4	120.00
N1—Hg1—N2	71.2 (3)	C5—C4—H4	120.00
Cl1^i^—Hg1—N1	82.2 (2)	N2—C6—H6	119.00
Cl1^i^—Hg1—N2	140.17 (19)	C5—C6—H6	119.00
Hg1—Cl1—Hg1^i^	100.10 (8)	C7—C8—H8	120.00
Hg1—N1—C1	125.9 (7)	C9—C8—H8	120.00
Hg1—N1—C5	116.6 (6)	C8—C9—H9	119.00
C1—N1—C5	117.5 (9)	C10—C9—H9	119.00
Hg1—N2—C6	112.2 (6)	C10—C11—H11	118.00
Hg1—N2—C7	125.9 (8)	C12—C11—H11	117.00
C6—N2—C7	121.8 (10)	C7—C12—H12	120.00
C10—N3—C13	117.2 (12)	C11—C12—H12	120.00
C10—N3—C15	114.4 (11)	N3—C13—H13A	108.00
C13—N3—C15	123.0 (11)	N3—C13—H13B	108.00
N1—C1—C2	123.0 (11)	C14—C13—H13A	108.00
C1—C2—C3	120.1 (11)	C14—C13—H13B	108.00
C2—C3—C4	117.3 (11)	H13A—C13—H13B	107.00
C3—C4—C5	120.9 (11)	C13—C14—H14A	109.00
N1—C5—C4	121.0 (9)	C13—C14—H14B	110.00
N1—C5—C6	118.1 (8)	C13—C14—H14C	110.00
C4—C5—C6	120.8 (9)	H14A—C14—H14B	109.00
N2—C6—C5	121.3 (8)	H14A—C14—H14C	109.00
N2—C7—C8	124.1 (13)	H14B—C14—H14C	110.00
N2—C7—C12	118.6 (14)	N3—C15—H15A	112.00
C8—C7—C12	117.2 (14)	N3—C15—H15B	112.00
C7—C8—C9	120.6 (12)	C16—C15—H15A	112.00
C8—C9—C10	122.8 (15)	C16—C15—H15B	112.00
N3—C10—C9	121.5 (14)	H15A—C15—H15B	110.00
N3—C10—C11	125.2 (15)	C15—C16—H16A	109.00
C9—C10—C11	113.3 (15)	C15—C16—H16B	109.00
C10—C11—C12	125.2 (16)	C15—C16—H16C	109.00
C7—C12—C11	120.7 (16)	H16A—C16—H16B	109.00
N3—C13—C14	118.6 (13)	H16A—C16—H16C	109.00
N3—C15—C16	98.2 (11)	H16B—C16—H16C	109.00
			
Cl2—Hg1—Cl1—Hg1^i^	−98.74 (12)	C7—N2—C6—C5	174.3 (10)
N1—Hg1—Cl1—Hg1^i^	69.9 (3)	Hg1—N2—C7—C8	−173.5 (10)
N2—Hg1—Cl1—Hg1^i^	139.95 (19)	Hg1—N2—C7—C12	4.6 (19)
Cl1^i^—Hg1—Cl1—Hg1^i^	0.00 (7)	C6—N2—C7—C8	3 (2)
Cl1—Hg1—N1—C1	−104.6 (8)	C6—N2—C7—C12	−178.5 (12)
Cl1—Hg1—N1—C5	76.4 (7)	C13—N3—C10—C9	−8 (2)
Cl2—Hg1—N1—C1	65.4 (9)	C13—N3—C10—C11	172.1 (16)
Cl2—Hg1—N1—C5	−113.6 (6)	C15—N3—C10—C9	−162.9 (15)
N2—Hg1—N1—C1	174.4 (9)	C15—N3—C10—C11	17 (2)
N2—Hg1—N1—C5	−4.6 (6)	C10—N3—C13—C14	91.1 (18)
Cl1^i^—Hg1—N1—C1	−35.7 (8)	C15—N3—C13—C14	−116.5 (16)
Cl1^i^—Hg1—N1—C5	145.3 (7)	C10—N3—C15—C16	−92.0 (14)
Cl1—Hg1—N2—C6	−125.6 (6)	C13—N3—C15—C16	114.9 (15)
Cl1—Hg1—N2—C7	51.6 (10)	N1—C1—C2—C3	−5.1 (18)
Cl2—Hg1—N2—C6	106.4 (6)	C1—C2—C3—C4	4.0 (17)
Cl2—Hg1—N2—C7	−76.5 (10)	C2—C3—C4—C5	−0.7 (16)
N1—Hg1—N2—C6	6.7 (6)	C3—C4—C5—N1	−1.7 (15)
N1—Hg1—N2—C7	−176.1 (10)	C3—C4—C5—C6	175.9 (9)
Cl1^i^—Hg1—N2—C6	−44.1 (7)	N1—C5—C6—N2	4.7 (13)
Cl1^i^—Hg1—N2—C7	133.1 (9)	C4—C5—C6—N2	−173.0 (9)
Cl1—Hg1—Cl1^i^—Hg1^i^	0.00 (9)	N2—C7—C8—C9	177.8 (14)
Cl2—Hg1—Cl1^i^—Hg1^i^	120.45 (11)	C12—C7—C8—C9	0 (2)
N1—Hg1—Cl1^i^—Hg1^i^	−135.1 (2)	N2—C7—C12—C11	−174.8 (14)
N2—Hg1—Cl1^i^—Hg1^i^	−87.4 (3)	C8—C7—C12—C11	3 (2)
Hg1—N1—C1—C2	−176.5 (9)	C7—C8—C9—C10	−1 (2)
C5—N1—C1—C2	2.6 (16)	C8—C9—C10—N3	179.4 (14)
Hg1—N1—C5—C4	179.9 (7)	C8—C9—C10—C11	−1 (2)
Hg1—N1—C5—C6	2.3 (10)	N3—C10—C11—C12	−176.2 (16)
C1—N1—C5—C4	0.8 (14)	C9—C10—C11—C12	4 (3)
C1—N1—C5—C6	−176.9 (9)	C10—C11—C12—C7	−6 (3)
Hg1—N2—C6—C5	−8.5 (10)		

Symmetry code: (i) −*x*+2, −*y*+1, −*z*+1.

### Hydrogen-bond geometry (Å, º)

**Table:**

*D*—H···*A*	*D*—H	H···*A*	*D*···*A*	*D*—H···*A*
C6—H6···Cl1^ii^	0.93	2.74	3.578 (9)	151
C1—H1···Cl1^i^	0.93	2.89	3.471 (12)	122
C1—H1···Cl2^iii^	0.93	2.97	3.623 (11)	129

Symmetry codes: (i) −*x*+2, −*y*+1, −*z*+1; (ii) −*x*+1, −*y*+1, −*z*+1; (iii) −*x*+2, −*y*+2, −*z*+1.
